# Data set on optimization of ethyl ester production from sapota seed oil

**DOI:** 10.1016/j.dib.2019.104388

**Published:** 2019-08-12

**Authors:** R. Sathish Kumar, S. Sivakumar, A. Joshuva, G. Deenadayalan, R. Vishnuvardhan

**Affiliations:** a Department of Automobile Engineering, Hindustan Institute of Technology and Science, Chennai, 603103, Tamil Nadu, India; b Department of Mechanical Engineering, Hindustan Institute of Technology and Science, Chennai, 603103, Tamil Nadu, India; c Department of Mechatronics Engineering, Sri Krishna College of Engineering and Technology, Coimbatore, 641008, Tamil Nadu, India

**Keywords:** Sapota seed oil, Optimization, Ethanolysis, Ethyl ester, Transesterification

## Abstract

This article presents the data set of experimental investigation on extraction, characterization, and optimization of ethyl ester yield from sapota seed oil. The seeds were collected, dried and shells were removed. Oil was extracted by mini wooden cold press oil extraction machine and found 26% oil content. The raw oil was characterized, fatty acid contents and physicochemical properties were estimated. The ethyl ester yield was optimized using full factorial experimental design. Three key factors were selected with three levels each. 27 experiments were conducted with three trials of each experiment. The physicochemical properties of the sapota seed oil ethyl ester were determined based on the ASTM standards and data was also presented in this data article.

**Table:** Specifications Table

Subject area	Renewable energy, Alternative fuel
More specific subject area	Biodiesel, Ethyl ester
Type of data	Raw Data
How data was acquired	Transesterification setup with magnetic stirrer with hot plate (Remi, India), Thermometer, weighing machine (ATKO scales Pvt. Ltd. India)
Data format	Raw and tabulated data
Experimental factors	Yield of ethyl ester, physicochemical properties of raw oil and its ethyl ester
Experimental features	Optimization of sapota seed oil ethyl ester process parameters
Data source location	Fuels and lubricants laboratory, Department of Automobile Engineering, Hindustan Institute of Technology and Science, Chennai, Tamilnadu, India.
Data accessibility	Data is with this article

**Table:** 

**Value of the Data** • This data provides the experimental results of transesterification process of the new biodiesel resource sapota seed oil with ethyl alcohol • New researchers and small scale biodiesel manufactures can use these experimental data • The optimized data for ethyl ester production from sapota seed oil can be used directly from this data article in future

## 1 Data

Sapota (*Manilkara Zapota (L.)*) seed oil is one of the non-edible unutilized third generation biodiesel resource. The oil content in the sapota seed is around 25%–30% of the weight of the seed. Methyl ester production from sapota oil has been optimized and engine performance, combustion and emission characteristics were also tested and reported in author's previous research articles [1], [2], [3]. The ethyl ester production from the sapota seed oil has not yet been attempted, hence this article is reporting the data set related to the optimization of ethyl ester production with three key parameters which are molar ratio of ethanol to oil, catalyst amount, and process temperature. The fatty acid composition and physicochemical properties of the sapota seed oil are presented in Table 1, Table 2, respectively. The chosen experimental factors and there levels are given in Table 3. The full factorial experimental design is used in this study. The detailed experimental design and their results are given in Table 4. Also the physicochemical properties of the sapota seed oil ethyl ester (SSOEE) are estimated and reported in Table 5 in this data article.

**Table 1:** Fatty acid composition of sapota seed oil.

Fatty acids	Content (%)	Molecular Weight (g/mol)
Palmitic acid (C16:0)	13.27	256.4
Stearic acid (C18:0)	2.80	284.5
Oleic acid (C18:1)	64.15	282.5
Linoleic acid (C18:2)	17.92	280.5
Linolenic acid (C18:3)	1.86	278.4

**Table 2:** Physicochemical properties of sapota seed oil.

Parameters	Values
Density at 15 °C (g/cm^3^)	0.887
Kinematic viscosity at 40 °C (mm^2^/s)	34.75
Free fatty acid (% FFA as oleic acid)	1.89
Acid value (mg KOH/g)	3.79
Iodine value (g Iodine/100 g)	65.02
Peroxide value (g/kg O_2_)	269.54
Color	Brownish yellow
Molecular weight (g/mol)	873.95
Percentage oil content in kernel (%)	23–30
Physical state at room temperature	Liquid
pH	3.5

**Table 3:** Experimental factors and their levels.

Levels	Experimental factors		
	Molar Ratio of Ethanol to Oil	Catalyst Amount (% wt)	Reaction Temperature (°C)
1	3:1	0.5%	50
2	6:1	1%	60 °C
3	9:1	1.5%	70 °C

**Table 4:** Experimental conditions and sapota seed oil ethyl ester yield (SSOEE).

Experiment Number	Molar Ratio of Ethanol to Oil	Catalyst Amount (% wt)	Reaction Temperature (°C)	SSOEE Yield (%)
1	3:1	0.5	50	68
2	3:1	0.5	60	69.5
3	3:1	0.5	70	70
4	3:1	1	50	74
5	3:1	1	60	76.3
6	3:1	1	70	78.2
7	3:1	1.5	50	64.3
8	3:1	1.5	60	66
9	3:1	1.5	70	65.4
10	6:1	0.5	50	81
11	6:1	0.5	60	83.1
12	6:1	0.5	70	84.8
13	6:1	1	50	87.2
14	6:1	1	60	89.8
15	6:1	1	70	93.2
16	6:1	1.5	50	78.7
17	6:1	1.5	60	81.2
18	6:1	1.5	70	82.8
19	9:1	0.5	50	73.4
20	9:1	0.5	60	76.8
21	9:1	0.5	70	69.8
22	9:1	1	50	73.2
23	9:1	1	60	76.4
24	9:1	1	70	79.8
25	9:1	1.5	50	82.8
26	9:1	1.5	60	86.4
27	9:1	1.5	70	81.6

**Table 5:** Physicochemical properties of sapota seed oil ethyl ester (SSOEE).

Properties	Units	Limits in the EN 14214 standard	SSOEE	Values for commercial Diesel for comparison purpose	Test method
Ester content	% (m/m)	Min 96.5	92.8	–	EN14103
Density at 15 °C	g/cm^3^	0.86–0.90	0.868	0.861	ASTM D4052
Kinematic viscosity	mm^2^/s	3.5–5	4.32	2.96	ASTM D445
Acid value	mg KOH/g	Max 0.50	0.19	0.18	ASTM D664
Iodine value	g iodine/100 g	Max 120	59.82	–	AOAC CD1-25
Pour point	°C	Max 0	−7	−12	ASTM D97
Flash point	°C	Min 120	169	48	ASTM D93
Heating value	MJ/kg	Min 35	39.15	44.8	ASTM D240
Cetane number	–	Min 51	51	51	ASTM D613
Sulphur content	mg/kg	Max 10	0	350	ASTM D5459

## 2 Experimental design, materials, and methods

### 2.1 Materials

**Fig. 1:**
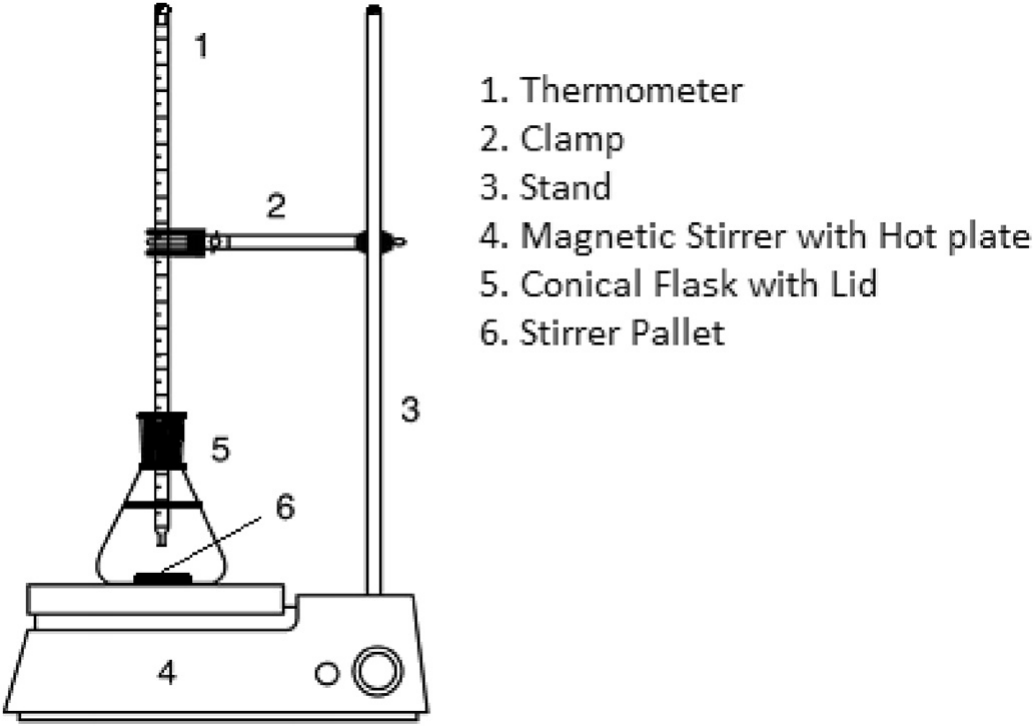
Schematic representation of experimental setup.

Sapota seeds were collected from the fruit forms located in southern states of India including Andrapradesh and Tamilnadu. The ethanol of 99% purity and KOH of 85% purity (procured from Pentagon chemicals manufacturer, Chennai, India) were used for transesterification process. The experimental setup used for transesterification process consists of a magnetic stirrer with hot plate (Remi, India), a thermometer of 0–100 °C range, 200 ml flat bottom conical flask with cork lid and 200 ml separating funnel. The schematic representation of experimental setup is shown in Fig. 1. A well-calibrated weighing machine of 0.001 g accuracy was used for accurate weighing of raw materials.

### 2.2 Oil extraction and characterization

The collected seeds were washed with water and dried at 60 °C in a seed drier for about 2 hours to evaporate the moisture content. The shells were removed manually and fresh kernels were crushed in a mini wooden cold press oil extraction machine. The raw oil was filtered and characterized. The fatty acid profile of the sapota seed oil was determined by Perkin-Elmer Clarus 500 gas chromatograph system coupled with a mass spectrometer. The fatty acid contents and their weight proportions are given in Table 1. Brookfield DV-II Proviscometer was used to measure the kinematic viscosity at 40 °C as per the procedure of ASTM D 445. The pour point and the cloud point were simultaneously estimated in accordance with ASTM D 5949 and ASTM D 5773, respectively using ANM brand, CPPA - 3C model number equipment. Flash point was measured using Pensky Martene open cup apparatus. Heating value was determined with the use of Parr – 6772 bomb calorimeter. Density at 15 °C was measured using a Rudolph DDM 2909 Automatic Density Meter. The measured physicochemical properties of sapota seed oil are listed in Table 2. The values of iodine number and cetane number were calculated as per the standards of ASTM D5554-15 and ASTM D613. The acid value was determined using a suitable titration with standardized KOH solution with phenolphthalein as the indicator [4], [5].

### 2.3 Transesterification process

The free fatty acid value of the sapota seed oil is 1.89% which is less than 2%, so that single step transesterification process was adopted to produce sapota seed oil ethyl ester. 50 (±0.1) grams of sapota oil is taken into the 200 ml conical flask and heated to the prefixed temperature (based on the experimental conditions) using hot plate. The oil was continuously stirred at 500 rpm using a magnetic stirrer and pellet, inbuilt with hot plate setup. Meanwhile, the required amount of ethanol and KOH (based on the molar ratio of ethanol to oil and percentage concentration of catalyst corresponding to the selected experimental condition) was measured exactly and mixed thoroughly to prepare ethoxide solution. Once the oil attained the required temperature, the prepared ethoxide solution was slowly poured into the conical flask. Each experiment was conducted for 90 minutes and repeated for three times and average value was taken [6].

### 2.4 Optimization of ethyl ester production

The full factorial optimization study was conducted for optimizing the yield of sapota oil ethyl ester. Three key factors were chosen, namely, ethanol to oil molar ratio, catalyst amount, and reaction temperature. Each factor was set to three levels, hence, total of 27 (L^P^ = 3^3^ = 27) experiments were conducted. The experimental factors and their levels are given in Table 3. The experimental conditions and average value of three trials of each experiment are given Table 4. The physicochemical properties of the biodiesel produced were estimated using ASTM standards and listed in Table 5.
